# Nationwide claims database analysis of treatment patterns, costs and survival of Japanese patients with diffuse large B-cell lymphoma

**DOI:** 10.1371/journal.pone.0237509

**Published:** 2020-08-18

**Authors:** Saaya Tsutsué, Kensei Tobinai, Jingbo Yi, Bruce Crawford

**Affiliations:** 1 Celgene KK, Tokyo, Japan; 2 National Cancer Center Hospital, Japan; 3 Syneos Health, Japan; University of Pennsylvania Perelman School of Medicine, UNITED STATES

## Abstract

Limited data are available regarding treatment patterns, healthcare resource utilization (HCRU), treatment costs and clinical outcomes for patients with diffuse large B-cell lymphoma (DLBCL) in Japan. This retrospective database study analyzed the Medical Data Vision database for DLBCL patients who received treatment during the identification period from October 1 2008 to December 31 2017. Among 6,965 eligible DLBCL patients, 5,541 patients (79.6%) received first-line (1L) rituximab (R)-based therapy, and then were gradually switched to chemotherapy without R in subsequent lines of therapy. In each treatment regimen, 1L treatment cost was the highest among all lines of therapy. The major cost drivers i.e. total direct medical costs until death or censoring across all regimens and lines of therapy were from the 1L regimen and inpatient costs. During the follow-up period, DLBCL patients who received a 1L R-CHOP regimen achieved the highest survival rate and longest time-to-next-treatment, with a relatively low mean treatment cost due to lower inpatient healthcare resource utilization and fewer lines of therapy compared to other 1L regimens. Our retrospective analysis of clinical practices in Japanese DLBCL patients demonstrated that 1L treatment and inpatient costs were major cost contributors and that the use of 1L R-CHOP was associated with better clinical outcomes at a relatively low mean treatment cost.

## Introduction

Non-Hodgkin lymphoma (NHL) is the most common hematologic malignancy worldwide, with the highest incidences reported in North America, Australia, New Zealand, and Western Europe [1]. In Japan, the incidence of NHL has been increasing steadily since 1993 and accounted for 39.6% of all hematologic malignancies in 2008 [2]. The most common NHL subtype in Japan between 2003 and 2008 was diffuse large B-cell lymphoma (DLBCL), which comprised 45.3% of all NHL, followed by follicular lymphoma (FL) (13.5%), and adult T-cell leukemia-lymphoma (ATLL) (8.3%). The distribution of NHL subtypes in Japan differs significantly from that in the United States, where DLBCL, chronic lymphocytic leukemia (CLL)/small lymphocytic lymphoma (SLL), and FL were the most common subtypes, accounting for 27.9%, 24.1%, and 15.1% of NHLs, respectively [2]. It is noteworthy that the above-mentioned common NHL subtypes are of B-cell origin with the exception of ATLL. The risk factors and therapy for NHL may vary greatly among subtypes and across regions [3].

The standard treatment for most subtypes of B-cell NHL is rituximab plus cyclophosphamide, doxorubicin, vincristine, and prednisone (R-CHOP) administered in six cycles given every three weeks. A retrospective population-based cancer registry study in Japan reported the 5-year relative survival rate for DLBCL patients to be 57% in 2003–2006, a 13% increase from 1993–1997 [4]. This increase may be due to the integration of rituximab into the previous chemotherapy-only combination. The R-CHOP regimen was approved for CD20-positive lymphomas, including DLBCL, in Japan in 2003, which confirmed the survival improvement based on changes in the treatment regimens [4–7]. Enhanced survival of DLBCL associated with rituximab (R)-based regimens was also reported in previous database studies in Ontario, Canada [8] and the United States [9]. In those earlier studies, overall survival (OS) rates were not comprehensively analyzed per detailed R-based treatment regimens, gender, and age groups.

The economic burden of DLBCL in the United States has been reported to be quite high; mean monthly cost per patient was 11,890 USD [10]. A previous US study that utilized Truven Health MarketScan claims database showed that hematopoietic stem cell transplant (SCT) and non-R-CHOP chemotherapy mostly impacted second-line (2L) and subsequent DLBCL treatment costs [11]. To date, there is no published literature that shows the economic burden, treatment-based OS, or time-to-next-treatment (TTNT) of DLBCL patients in Japan.

Using Japanese nationwide claims database, Medical Data Vision (MDV) database, in Japan, we first report here current treatment patterns (i.e., lines of therapies and proportion of patients on each therapy), healthcare resource utilization (HCRU), healthcare costs, OS, and TTNT for DLBCL patients per detailed treatment regimens (including multiple R-based regimens), gender, and age groups in routine clinical practice in Japan. The MDV database represents approximately 8% of the Japanese population, as of August 2019.

## Materials and methods

### Database and patient selection

Data for this retrospective claims study were analyzed from the MDV database, an administrative database of which comprised of anonymized inpatient and outpatient data from 382 Japanese hospitals, covering approximately 22% of acute phase hospitals (including 187 cancer therapeutic facilities) and 26.77 million patient records as of December, 2017. The identification period was October 1, 2008 through December 31 2017, with a look-back period of 6 months and a follow-up period of 12 months relative to the patient index date (defined as the first date of treatment for DLBCL during the identification period).

Patients were included if they were treated for DLBCL during the identification period and had a claim with an International Classification of Diseases (ICD)-10 diagnosis code of DLBCL (C83.3x, C84.6x, C84.7x, or C85.2x) on the index date or during the six-month look-back period. Patients were excluded if they did not have at least two claims (for any disorder) with one-year follow-up (one claim every six months) unless they died or did not have at least one claim (for any disorder) within a six-month look-back period.

### Clinical outcome measures and data analyses

The primary treatment pattern outcome measures included number of lines of therapy and proportion of patients who received each therapy, which included R-CHOP regimens, rituximab plus cyclophosphamide, vincristine, and prednisone (R-CVP) regimens, R + bendamustine regimens, R + other chemotherapy, R monotherapy, chemotherapy without R, other therapy without R (immunotherapy, targeted therapy and hormone therapy), and all other treatments classified under not otherwise specified (NOS) regimens. According to a phase II clinical study [12], R + bendamustine was suggested as a substitute treatment option for R-mini-CHOP therapy (R-CHOP therapy with reduced dose and cycles) for elderly patients. The clinical outcome measures of OS and TTNT Kaplan-Meier (KM) curve analyses were finally conducted for regimen groups with a cut-off value of ≥100 patients who received R-CHOP, R-CVP, R + other chemotherapy, or chemotherapy without R. Because patients may have received prior treatments not captured in the MDV database, the exact line number could not be determined. Therefore, we defined the first observed line as 1L (e.g., first-line [1L], second-line [2L]).

All drugs added within 30 days of initiating a new line of therapy were considered a part of the same regimen and line of therapy. A new line of therapy was defined as either a new drug added after 30 days of initiation or a 90-day gap between any treatment (whichever came first) advancing the line of therapy.

Measures of HCRU included the number of outpatient visits, number of hospitalizations, length of hospital stay, and number of emergency room visits during each line of therapy. In addition, direct medical costs were calculated during the treatment period and stratified by inpatient, outpatient, cancer drug costs, and other pharmacy costs. The actual costs were converted from Japanese yen (JPY) to United States dollars (USD) using the exchange rate as of January in each year assessed. One-way ANOVA was performed to evaluate any 1L treatment group differences in specific HCRU outcomes of interest. Univariate gamma GLM with log-link functions were used to evaluate any 1L treatment group differences in cost outcomes.

Exploratory measures included OS during the follow-up period, calculated for all patients and stratified by demographic factors (age and gender) and treatment regimen types. In addition, a modified TTNT during follow-up period was calculated from the start of 1L treatment and was stratified by age and gender. TTNT was defined as a composite outcome of subsequent lines of therapy or death. Patients without the TTNT outcome were censored at their last patient record or at end of the follow-up period, whichever came first.

Continuous variables that followed an approximately normal distribution were summarized using mean, standard deviation (SD), median, minimum, and maximum. Categorical variables were summarized using frequency and percentage. Multivariable logistic regression models were constructed with not receiving R in each line of therapy as the outcome variable and gender, age group, each Charlson Comorbidity, and receiving R in any prior line as the covariates to evaluate factors associated with not receiving R-based therapies. For OS and TTNT analyses, Kaplan-Meier survival curves and corresponding median and 95% confidence intervals were generated for all patients and stratified by 1L treatment group. All data analyses were performed using SAS^®^ software version 9.4 or higher.

## Results

**Fig 1:**
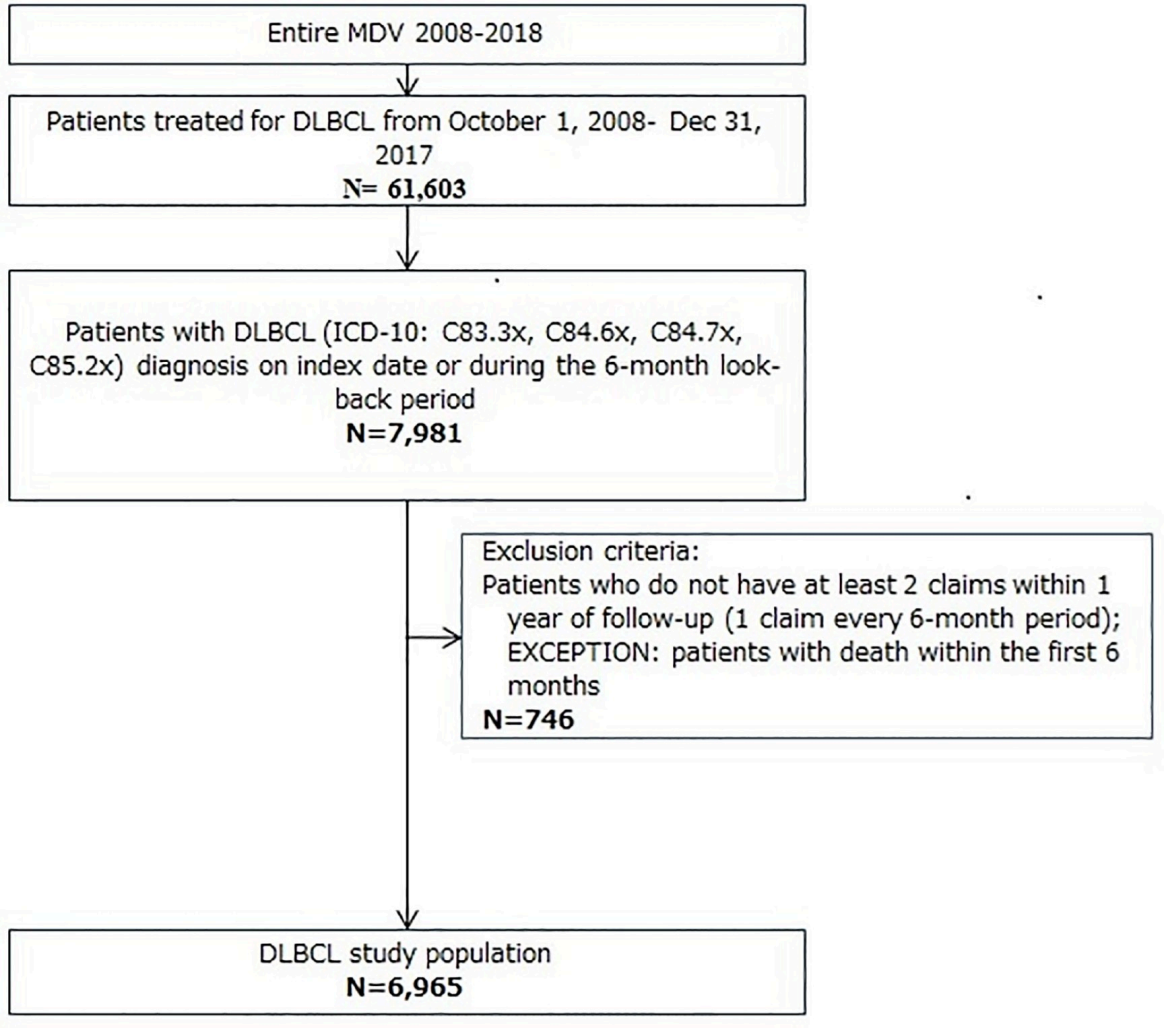
Patient selection for inclusion in analysis (identification period October 1 2008 to December 31 2017). DLBCL: diffuse large B-cell lymphoma; ICD-10: International Classification of Diseases Revision 10.

During the study identification period from October 1 2008 through December 31 2017, a total of 7,981 patients in the MDV database were treated for DLBCL with a DLBCL diagnosis on the index date or during the six-month look-back period (Fig 1). After applying the remaining criteria, 6,965 patients met the inclusion criteria.

Patient baseline characteristics are shown in Table 1. The mean age for patients with DLBCL included in the analysis was 69.9 (SD: 12.7) years, with a range of 5.0 to 99.0 years. The majority (n = 5,023; 72.1%) were ≥65 years of age and 1,597 (22.9%) were ≥80 years of age. Approximately half of patients (54.1%) were male. The median follow-up time was 912 days (inter-quartile range, 408–1,391). A relatively high comorbidity burden was observed during the look-back period, probably due to the elderly cohort population; the most common comorbidities were prior or concurrent primary cancers (n = 1,437; 20.6%), mild liver disease (n = 1,295; 18.6%), congestive heart failure (n = 1,072; 15.4%) and chronic pulmonary disease (n = 1,008; 14.5%).

**Table 1:** Patient demographics and baseline characteristics.

	Patients with DLBCL	
	N = 6,965	
Mean age in years (SD)	69.9	12.7
Minimum, maximum	5.0	99.0
Age in years, n (%)		
0–17	16	0.23%
18–39	186	2.67%
40–44	105	1.51%
45–49	172	2.47%
50–54	263	3.78%
55–59	476	6.83%
60–64	724	10.40%
65–69	1,064	15.28%
70–74	1,117	16.04%
75–79	1,245	17.88%
80–84	993	14.26%
85+	604	8.67%
Gender, n (%)		
Female	3,198	45.9%
Male	3,767	54.1%
Baseline CCI, n (%)		
0	69	1.0%
1	4	0.1%
2	3,358	48.2%
3	751	10.8%
4+	2,783	40.0%
Follow-up time in days (from index date until death or last patient record)		
Mean (SD)	936.6	598.0
Median (Q1, Q3)	912.0	408.0, 1391.0
Minimum, maximum	1.0	3550.0
Other prior or concurrent primary cancer (C00-C96, except C77-C89), n (%)		
Yes	1,437	20.6%
No	5,528	79.4%
Death within MDV during the identification or follow-up period, n (%)		
Yes	1,819	26.1%
No	5,146	73.9%

CCI: Charlson Comorbidity Index; MDV: Medical Data Vision; SD: standard deviation.

### Treatment characterization

Treatment patterns of DLBCL therapy including R-CHOP, R-CVP, R + bendamustine, R + other chemotherapy, R monotherapy and chemotherapy without R are shown in Table 2.

**Table 2:** DLBCL treatment patterns.

Treatment Patterns	Patients with DLBCL	
	N = 6,965	
1L regimen, n (%)		
R-CHOP	3,343	48.0%
R-CVP	1,160	16.7%
R + bendamustine	30	0.4%
R + other chemotherapy or R + dexamethasone	955	13.7%
R monotherapy	53	0.8%
Chemotherapy without R	837	12.0%
Other (immunotherapy, targeted therapy, hormone) without R	32	0.5%
Others	555	8.0%
Subsequent regimen 2L, n (%)		
R-CHOP	425	12.5%
R-CVP	200	5.9%
R + bendamustine	51	1.5%
R + other chemotherapy or R + dexamethasone	934	27.4%
R monotherapy	89	2.6%
Chemotherapy without R	947	27.8%
Other (immunotherapy, targeted therapy, hormone) without R	90	2.6%
Others	668	19.6%
Subsequent regimen 3L, n (%)		
R-CHOP	115	6.3%
R-CVP	38	2.1%
R + bendamustine	39	2.1%
R + other chemotherapy or R + dexamethasone	516	28.2%
R monotherapy	54	2.9%
Chemotherapy without R	652	35.6%
Other (immunotherapy, targeted therapy, hormone) without R	63	3.4%
Others	355	19.4%
Subsequent regimen 4L, n (%)		
R-CHOP	41	3.9%
R-CVP	13	1.2%
R + bendamustine	28	2.7%
R + other chemotherapy^a^ or R + dexamethasone	300	28.7%
R monotherapy	30	2.9%
Chemotherapy without R	369	35.3%
Other (immunotherapy, targeted therapy, hormone) without R	59	5.7%
Others	204	19.5%
Subsequent regimen 5L, n (%)		
R-CHOP	10	1.7%
R-CVP	13	2.2%
R + bendamustine	10	1.7%
R + other chemotherapy or R + dexamethasone	127	21.9%
R monotherapy	18	3.1%
Chemotherapy without R	262	45.1%
Other (immunotherapy, targeted therapy, hormone) without R	45	7.7%
Others	96	16.5%
Number of lines, n (%)		
1	3,561	51.1%
2	1,572	22.6%
3	788	11.3%
4	463	6.6%
5	264	3.8%
6+	317	4.6%
Duration of 1L regimen, days		
Mean (SD)	121.6	133.1
Median (Q1, Q3)	113.0	51.0, 154.0
Minimum, maximum	1.0	2045.0
Patients using R maintenance therapy^a^, n (%)	25	0.5%
Patients who received stem cell transplant across all lines of therapy, n (%)	284	4.1%
*Age distribution of stem cell recipients*, *n (%)*		
<50	64	22.5%
50–64	170	59.9%
65–79	50	17.6%
80–84	0	0.0%
85+	0	0.0%
Patients who received radiation therapy across all lines of therapy, n (%)	1,518	21.8%
During 1L (prior to 2L)	905	13.0%
During 2L (prior to 3L)	406	5.8%
During 3L (prior to 4L)	196	2.8%
During 4L (prior to 5L)	117	1.7%
During 5L (prior to 6L+)	114	1.6%

1L: first-line; 2L: second-line; 3L: third-line; 4L: fourth-line; 5L: fifth-line; DLBCL: diffuse large B-cell lymphoma; R: rituximab; R-CHOP: rituximab plus cyclophosphamide, doxorubicin, vincristine, and prednisone/prednisolone; R-CVP: rituximab plus cyclophosphamide, vincristine, and prednisone; SD: standard deviation.

^a^ Patients who received R-based 1L regimen only.

Nearly half of patients (n = 3,343; 48.0%) received R-CHOP as the 1L regimen. A total of 2,145 patients (30.8%) received R + other therapy, including 1,160 (16.7%) patients who received R-CVP therapy. There were 869 patients (12.5%) who received chemotherapy or other therapy without R and 53 patients (0.8%) received R monotherapy. The most frequent 1L regimen received by patients 66 years and older was R-CHOP (n = 1,987, 41.2%), followed by R-CVP (n = 1053, 21.8%). In contrast, a higher proportion of younger patients received R-CHOP in 1L (n = 1,356, 63.4%), followed by R + other chemotherapy (n = 278, 13.0%) (S2 Table). While the proportion of patients on other regimens remained steady, the proportion of R-CHOP gradually decreased while the proportion of R-CVP increased with each advanced line of therapy. The mean (SD) duration of the 1L treatment was 121.6 (133.1) days (range, 1.0–2,045.0 days).

The maximum number of lines of therapy analyzed was 5 from the 1L treatment to the end of the patients’ follow-up period, which are shown in Table 2. For each subsequent therapy, more patients received R-CVP or chemotherapy without R than other regimen categories. Approximately half of patients (n = 3,561; 51.1%) received only one line of therapy through the end of their patient record; 317 patients (4.6%) received 6 or more lines of therapy. Across all lines of therapy, 284 (4.1%) patients received SCT and 1,518 (21.8%) received radiation therapy. The percentage of patients who received radiation therapy was 13.0% during 1L, decreasing as line of therapy proceeded. The purpose of radiation therapy might be interpreted as a curative intent in 1L, but a palliative intent in the further lines.

### Healthcare resource utilization and costs

**Fig 2:**
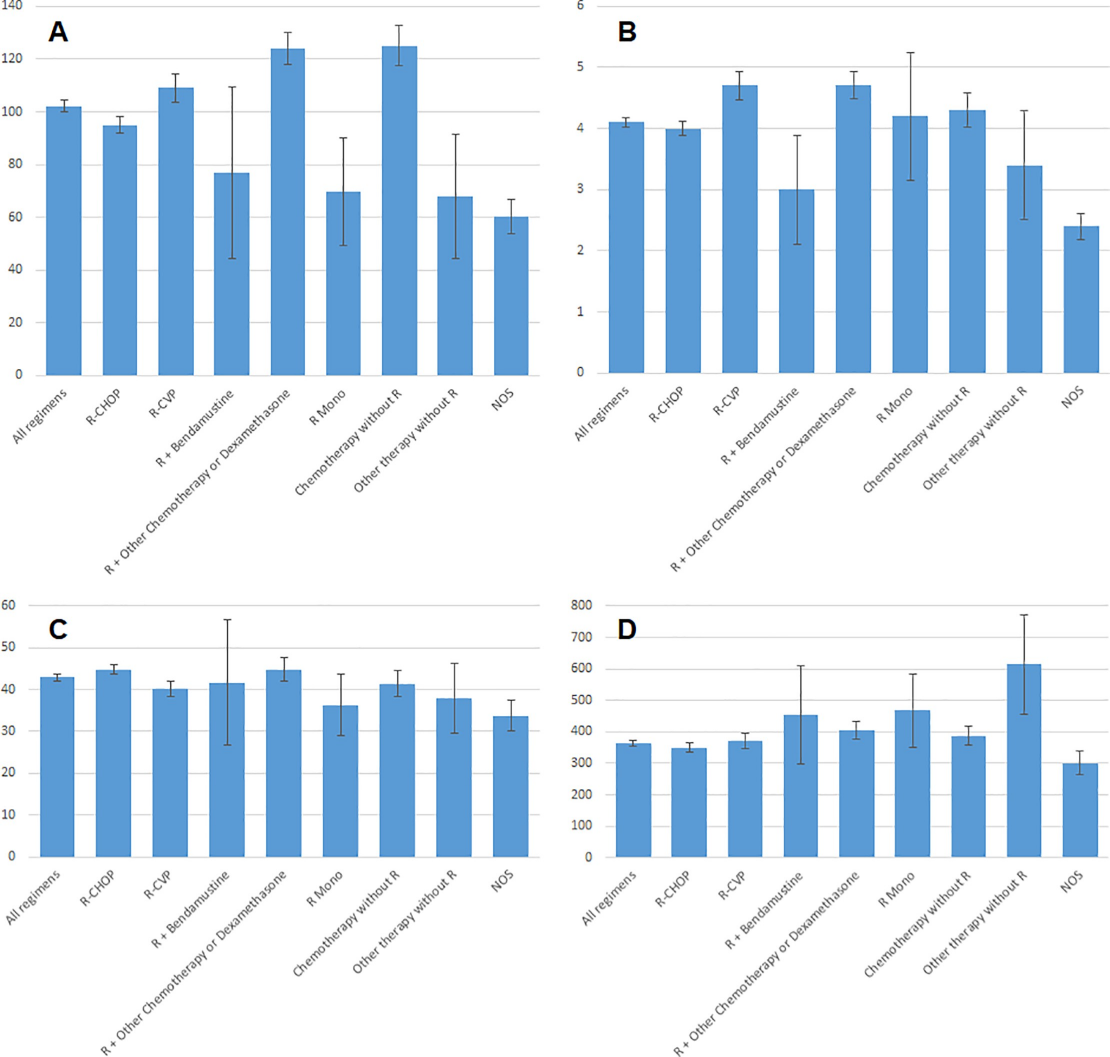
DLBCL-related HCRU by 1L treatment. (A) Average total days of inpatient stay (ANOVA, p<0.0001). (B) Average number of hospitalizations (p<0.0001). (C) Average number of outpatient visits (p<0.0001). (D) Average overall treatment duration (p<0.0001). Error bars indicate 95% confidence limit.

HCRU during the follow-up period after 1L and during each line of therapy are shown in Fig 2. Patients who received R-CVP and R + other therapy in 1L experienced more hospitalizations (mean of 4.7 for both groups) during the follow-up period compared to other regimens. Despite slightly fewer mean number of hospitalizations (4.3), patients who received chemotherapy without R had the longest mean length of stay (LOS; 125.0 days) during the follow-up period, followed by R + other therapy (124.0 days) and R-CVP (109.0 days). Patients who received R-CHOP regimen as 1L underwent fewer hospitalizations (mean of 4.0) and had a shorter LOS (mean of 95.0 days) than those who received R-CVP, R + other therapy and chemotherapy without R during the follow-up period. When only 1L treatment period was taken into consideration, mean LOS during R-CVP (68.6 days) and R + other therapy (66.5 days) were longer than those during R-CHOP (58.5 days) and chemotherapy without R (52.3 days). (S3 Table)

Patients who received R-CHOP as 1L experienced the shortest total treatment duration (350.1 days) and fewer number of lines of therapy (1.9) except for R + bendamustine and NOS (1.9 and 1.7, respectively). In contrast, patients who underwent other types of therapy without R as 1L experienced the longest total treatment period (614.8 days) and the largest number of lines of therapy (3.4), respectively, followed by chemotherapy without R (with 2.9 lines of therapy).

**Fig 3:**
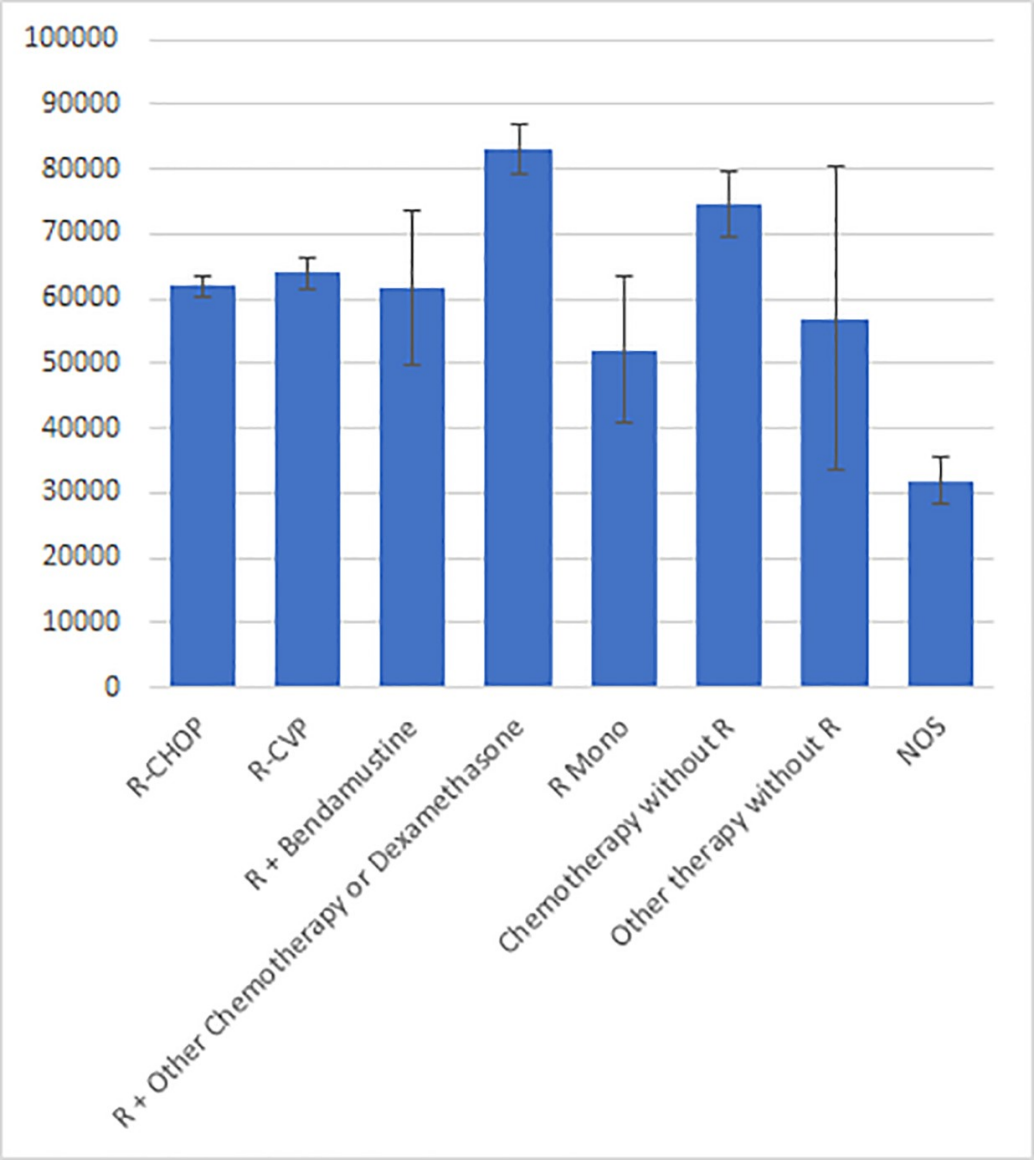
Total cost during follow-up. Cost in USD; error bars indicate 95% confidence limit.

Direct costs during the follow-up period after 1L treatment are shown in Fig 3. The mean total cost during the follow-up period for all 6,965 patients was 64,180.43 USD (SD, 52,945.20; range, 317.33–738,221.48), and were higher with R + chemotherapy and chemotherapy without R compared to other therapies. The overall total cost was significantly different between treatment groups (p<0.001). Mean 1L costs were highest with R + bendamustine, R-CVP, and R + other chemotherapy, compared to lower costs with R monotherapy and chemotherapy without R (S4 Table).

**Fig 4:**
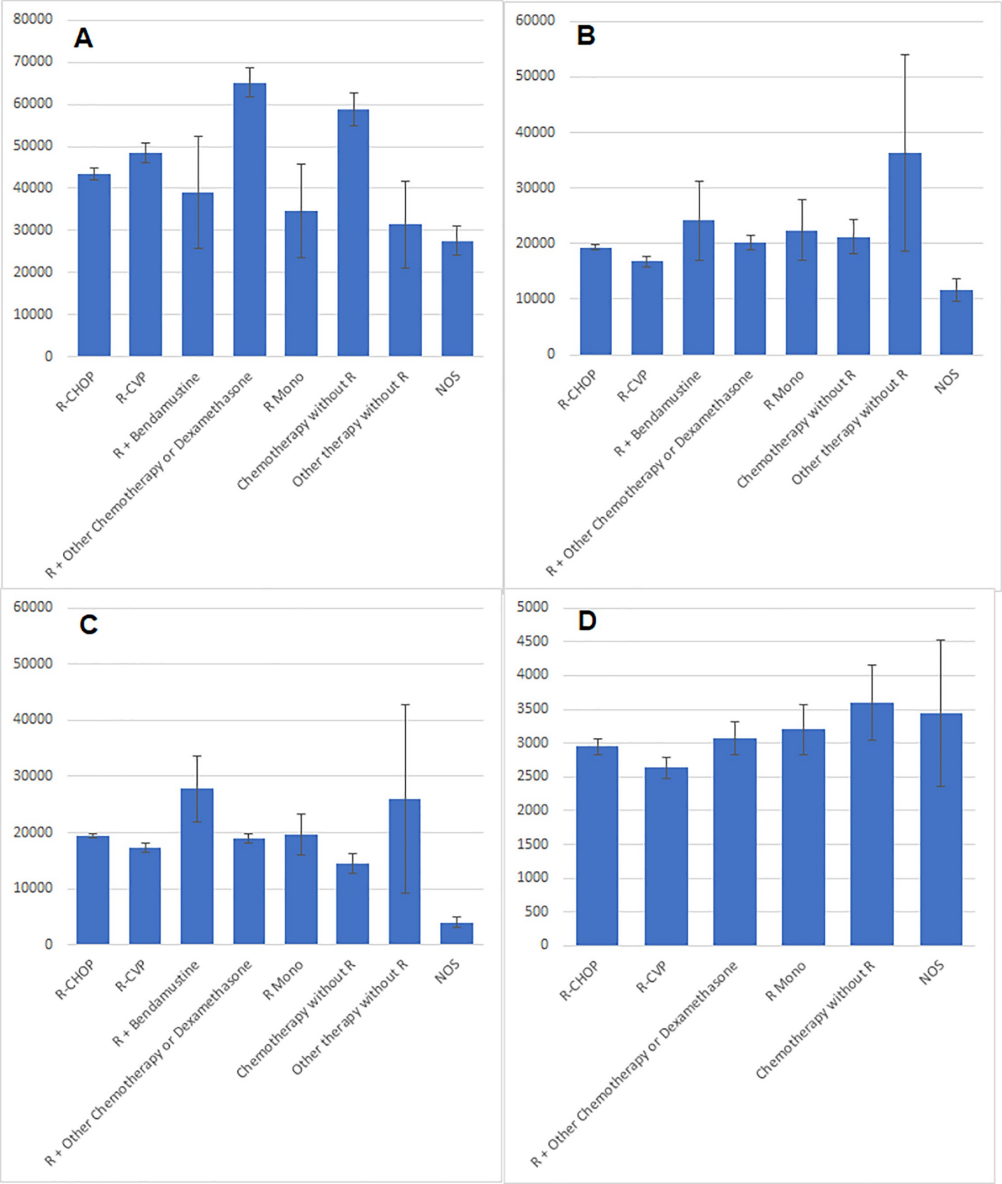
DLBCL-related costs by 1L treatment (USD; error bars indicate 95% confidence limit). (A) Average inpatient cost (p<0.0001). (B) Average outpatient cost (p<0.0001). (C) Average total cancer treatment cost (p<0.0001). (D) Average stem cell transplant cost (p = 0.0010; no data available for R + bendamustine and other therapy without R).

Fig 4 presents the cost contributors by 1L regimen. A majority of the overall and per-treatment line costs were due to inpatient costs; mean inpatient costs (n = 6,821) were 47,903.08 USD (SD, 47,497.30; range, 247.43–488,296.86). Total and per-regimen costs of regimens were highest in 1L (S4 Table).

### Overall survival

**Fig 5:**
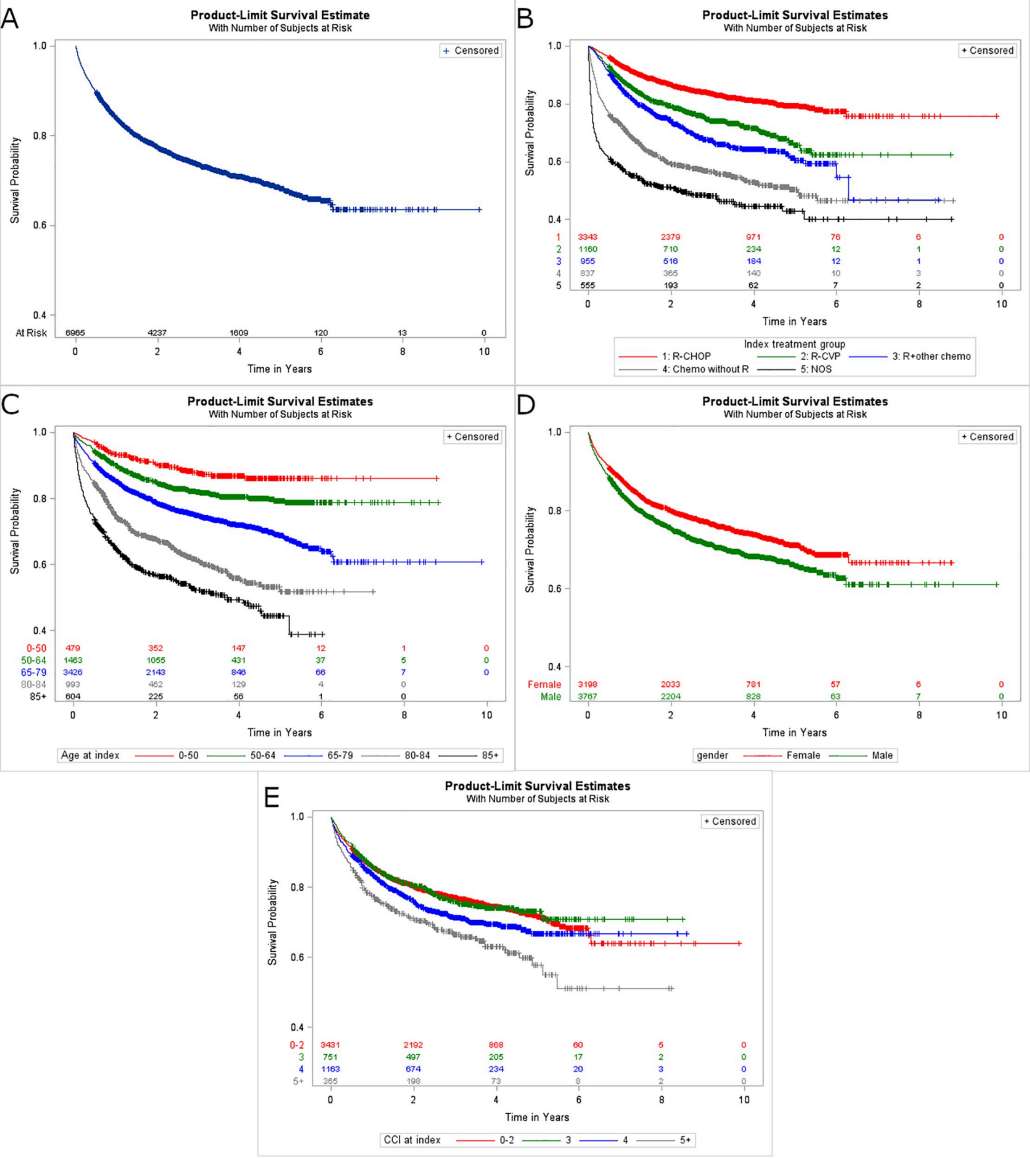
Kaplan-Meier plots for overall survival. (A) Overall survival and stratified by (B) 1L regimen group, (C) patient age, (D) gender, and (E) CCI score. CCI: Charlson Comorbidity Index; OS: overall survival; R: rituximab; R-CHOP: rituximab plus cyclophosphamide, doxorubicin, vincristine, and prednisone.

Kaplan-Meier curves for OS outcomes are shown in Fig 5. Overall, there were 1,819 deaths (26.1% of patients) within the follow-up period across all 1L treatment regimens (Fig 5A). The survival proportion at the end of the follow-up period was 73.9% for all patients. When stratified by 1L regimen, the highest overall survival proportion was observed in the R-CHOP group and R + bendamustine, followed by R-CVP (83.3%, 83.3%, 74.8%, respectively); the KM representation of the conditional survival probabilities at each time point are shown in Fig 5B. There was an increase in the risk of death with increasing age (Fig 5C). Overall, the number of deaths increased across each higher age grouping, from 56 deaths (11.7%) in patients <50 years of age to 271 deaths (44.9%) in patients ≥85 years of age. A slightly higher risk of death was observed in males compared to females (Fig 5D). A positive association was observed between increase of higher CCI score and risk of death (Fig 5E).

### Time to next treatment

**Fig 6:**
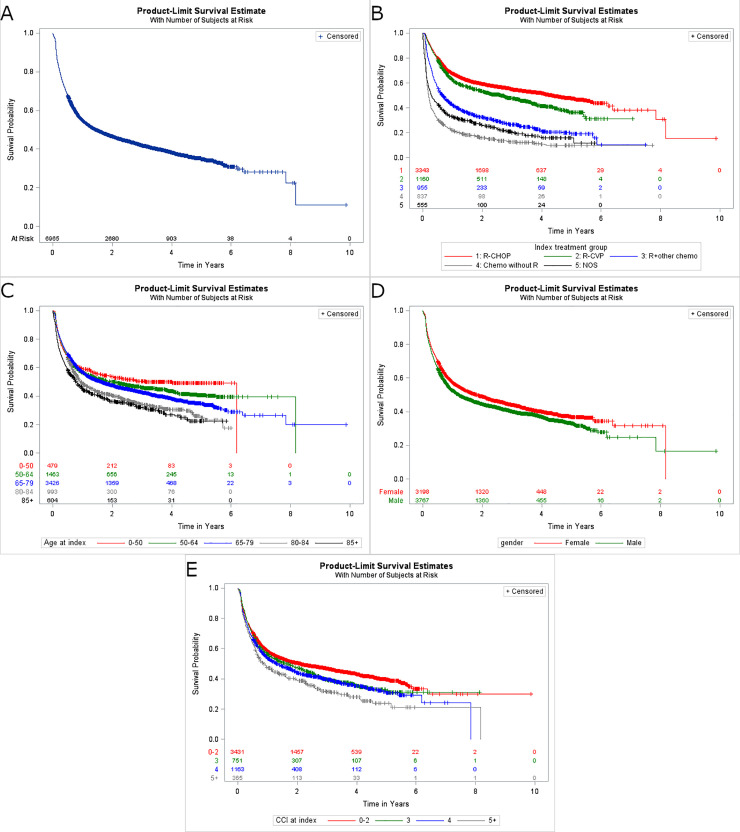
Kaplan-Meier plots for time to next treatment. (A) Overall TTNT and stratified by (B) 1L regimen group, (C) patient age, (D) gender, and (E) CCI score. CCI: Charlson Comorbidity Index; R: rituximab; R-CHOP: rituximab plus cyclophosphamide, doxorubicin, vincristine, and prednisone; TTNT: time to next treatment.

Outcomes for Time to Next Treatment (TTNT) are shown in Fig 6. Overall, nearly half of all patients (n = 3,404; 48.9%) received their next treatment within the follow up period; 4,063 patients (58.3%) had either received a next treatment or died. Median TTNT was 520 days (95% CI, 473–586; Fig 6A). When stratified by 1L regimen, the lowest event-free (defined as no next treatment or death) proportion at the end of follow-up period among specified treatment groups was observed in chemotherapy without R (14.20%), while the highest event-free probability was R-CHOP, followed by R-CVP and other types of therapy without R (54.0%, 47.1%, 40.6%, respectively). When stratified by age, the K-M median TTNT decreased with increasing age (from 1,145 days [95% CI, 616 to 2,229] for patients <50 years of age to 283 days [95% CI, 224–352] for patients ≥85 years of age; Fig 6C). When stratified by gender, median TTNT was higher for female patients compared to male patients (652 days [95% CI: 560–765] vs 436 days [95% CI: 391–498]; Fig 6D). When we computed median TTNT by CCI score the TTNT was shorter for higher CCI scores (Fig 6E).

### Odds of not receiving R-based regimens

To clarify clinical or patient baseline attributes that may have contribute to patient not receiving R-based regimens, the odds of not receiving R-based regimens was calculated using multivariable logistic regression analysis. In Table 3, our results show that, compared to patients aged <66 years, patients aged 81–85 or over 85 years were significantly less likely to receive R-based regimens in 1L (p<0.05).

**Table 3:** Odds of not receiving R-based regimens in 1L.

Characteristics	n (%)	OR (Lower CI-Upper CI)
Number of patients who did not receive R during specified line (%)	1,424 (20.45%)	
Age		
<66*	380 (26.69%)	
66–70	204 (14.33%)	1.06 (0.87–1.28)
71–75	199 (13.97%)	0.91 (0.75–1.11)
76–80	261 (18.33%)	1.15 (0.96–1.37)
81–85	211 (14.82%)	*1*.*32 (1*.*09–1*.*61)*
Over 85	169 (11.87%)	*2*.*52 (2*.*01–3*.*16)*
Gender		
Male*	783 (54.99%)	
Female	641 (45.01%)	0.9 (0.8–1.02)
Charlson comorbidities**		
Congestive heart failure	273 (19.17%)	*1*.*22 (1*.*04–1*.*43)*
Dementia	52 (3.65%)	*2*.*21 (1*.*52–3*.*22)*
Chronic pulmonary disease	219 (15.38%)	1.04 (0.88–1.23)
Rheumatologic disease	84 (5.9%)	*1*.*98 (1*.*5–2*.*6)*
Mild liver disease	299 (21%)	*1*.*18 (1*.*01–1*.*37)*
Diabetes with chronic complications	40 (2.81%)	0.85 (0.59–1.21)
Hemiplegia or paraplegia	44 (3.09%)	*4*.*98 (3*.*18–7*.*81)*
Renal disease	84 (5.9%)	*1*.*61 (1*.*22–2*.*13)*
Any malignancy, including lymphoma and leukemia	1,393 (97.82%)	*0*.*52 (0*.*34–0*.*8)*
Moderate or severe liver disease	15 (1.05%)	*1*.*99 (1*.*04–3*.*81)*
Metastatic solid tumor	209 (14.68%)	*1*.*26 (1*.*06–1*.*49)*
HIV	5 (0.35%)	2.67 (0.86–8.33)

* Reference group

** For Charlson comorbidities, reference group was "No"

OR with P < 0.05 is written in *italics*.

In addition, patients with Charlson comorbidities at baseline including congestive heart failure, dementia, rheumatologic disease, mild liver disease, hemiplegia/paraplegia, renal disease, moderate to severe liver disease, and metastatic solid tumors were more likely not to receive R-based regimens in 1L (p<0.05). For all lines of therapy from 2L to 5L, receiving R in any prior lines was inversely associated with not receiving R in the current line (p<0.05) (Table 4).

**Table 4:** Odds of not receiving R-based regimens after receiving R-based regimens in any prior line(s).

Receiving R in any prior lines	2L	3L	4L	5L
n (%)	1,268 (74.37%)	878 (82.06%)	532 (84.18%)	338 (83.87%)
OR* (Lower CI-Upper CI)	*0*.*81 (0*.*69–0*.*95)*	*0*.*18 (0*.*12–0*.*27)*	*0*.*09 (0*.*04–0*.*2)*	*0*.*08 (0*.*02–0*.*27)*

* OR with P < 0.05 is written in *italic****s***; **R**eference group = not receiving R in prior lines

## Discussion

To our knowledge, this is the first study to evaluate lines of therapy treatment patterns, clinical outcomes, and HCRU among patients with DLBCL in Japan. Guidelines from the National Comprehensive Cancer Network (NCCN), European Society for Medical Oncology (ESMO) and British Society for Haematology (BCSH) recommend an R-based regimen including R-CHOP and R-CVP as 1L treatment for DLBCL. Consistent with that recommendation, nearly 80% of Japanese DLBCL patients received an R-based regimen as 1L in this study. Although not directly comparable, the observed R-based treatment frequency was lower than that of an US claims database study (95.9%) [10] and an US SEER Medicare Linked Database study (93.1% during 2010–2014) [15].

Age and comorbidities are key determinants of deciding whether to choose R-based regimens for eldery DLBCL patients [13]. Our multivariable logistic regression analysis, indeed, demonstrates that age over 80 and the presence of some comorbidities are associated with not choosing an R-based therapy. Toxicity intolerance and virus reactivation were reported to be associated with R-based regimens in some DLBCL patients with organ dysfunction disorder or other comorbidities [14]. The percentage of patients over 80 is about 23% in our study, which is lower than that in the US SEER study (35.9%). On the other hand, the CCI was also higher in our study (score 3+: 51.6%) compared to the US SEER study (score 3+: 26.9%). This is likely due to the differential in time period from which comorbidities are evaluated. The US SEER study evaluated comorbidities from initial diagnosis; however, because our study evaluated comorbidities from treatment initiation, nearly all patients by definition had a cancer diagnosis (DLBCL) at baseline. Despite these different definitions, logistic regression models suggest that the presence of several comorbidities at baseline might be a reason for less frequent use of R-based therapy in this study.

Earlier research that evaluating treatment patterns of DLBCL patients up to 3L in the US reported gradual treatment transition from R-based regimens towards chemotherapy without R as lines of therapy increased [10], which was generally consistent with our results in Japan. In our study, patients who did not receive an R-based regimen in advanced lines (2L+) of therapy were less likely to have received R-based therapy in 1L. This suggests that patients who were ineligible to receive more potent R-based therapy in 1L were less likely to be cured and thus more likely to proceed to advanced lines. The occurrence of R resistance could also be associated with the decreasing tendency of R usage in further lines of therapy, which cannot be evaluated with the current dataset.

Our analysis further showed that R-CHOP was the most preferred 1L treatment. However, it was only chosen for 1.7% of patients in fifth line (5L) regimens. In contrast, R-CVP, R-bendamustine, and other R-based chemotherapy regimens were consistently chosen across all lines of therapy that we investigated, accounting for over 25.8% of patients overall, even in 5L. This study using real-world data demonstrated a small portion of patients with R-CVP or R-bendamustine as 1L. This may be a limitation of the algorithm used in the dataset to classify index regimen as 1L regimen instead of more advanced lines. However, it is also a possible reflection of real-world practice. As doxorubicin-related cardiotoxicity is also a well-documented clinical concern for physicians, patients with high risk of cardiotoxicity may not be eligible for R-CHOP and are based on other cardio-protective strategies [15–19]. Długosz-Danecka et al. concluded that the effectiveness of various cardio-protective strategies is heterogeneous and unconfirmed in clinical practice [20]. Therefore, it is plausible that many of the 15.4% patients who had congestive heart failure at baseline in this study were considered for such cardio-protective strategies.

In a Canadian study [21], autologous SCT and hospitalization contributed the most to direct costs in 2L+. However, in this study of Japan, inpatient costs contributed to most of the total cost throughout each line of therapy, suggesting that regimens that incur less hospitalizations and shorter lengths of stay may reduce both patient’s economic burden and the national healthcare budget. A high proportion of DLBCL inpatient cost was also noted by Costa et al. [22] in a Canadian real-world cost analysis. Another major cost driver was either longer total duration or increased lines of therapy. With R-CHOP, the length of stay, total treatment duration, and total lines of therapy that patients received were relatively short, leading to lower costs than those of R-CVP, R + bendamustine, R + other chemotherapy, and chemotherapy without R. Although the cost of 1L chemotherapy without R was much lower cost compared to R-CHOP regimen, the total cost was higher. This may be partially explained by the higher average number of treatment lines experienced by patients whose 1L treatment was chemotherapy without R, and the possibility for patients to proceed to R-based therapies in subsequent lines.

Stratified Kaplan-Meier survival analyses showed worse survival with increasing age, male gender, and use of chemotherapy without R. Such trends were also observed in an US SEER registry study [23]. Association of R-based regimens with higher survival rates has been suggested in earlier reports [4,8,9]. Our results further demonstrate that the survival rate was highest with R-CHOP, followed by R-CVP, R + other chemotherapy and chemotherapy without R, which were also supported by TTNT outcomes, reflecting the treatment modality of patients in another real-world setting study [24].

There were several limitations in this database study. Patients’ treatment history before their first visit was not traceable due to limited accessibility to prior records. Although the algorithm used to identify each line of therapy is clinically meaningful and had been used in prior database studies, its reliability to classify the treatment regimen in each line of therapy is presumed to decrease for later lines of therapy. This analysis classified patient’s 1L regimen based on their first treatment during the identification period and required at least 6 months of look-back with a DLBCL diagnosis. Most patients were assumed to be treatment naïve, and based on the 1L regimen distribution, the results were consistent with prior literature [23]. Another limitation was that mortality data were captured only through patient’s hospital discharge records, making OS results less robust. Although mortality was not fully captured, missing data were expected to be random and therefore comparison between groups was likely still meaningful. Patients without a death record were censored at their last claim, and therefore did not contribute to follow-up time beyond their last record.

In conclusion, this study found that R-based regimens were frequently chosen for 1L treatment in DLBCL in Japan, but gradually were switched to chemotherapy without R in higher lines of therapy. The main cost drivers were type of the 1L regimen and inpatient costs. During the entire follow-up period, DLBCL patients receiving an R-CHOP regimen achieved the highest survival rate and longest median TTNT with lower inpatient HCRU, resulting in a relatively low mean total treatment cost.

## Supporting information

S1 Table Additional baseline characteristics for the patient cohort. ^a^Each comorbidity was evaluated at 12 months prior to treatment initiation; because the cohort by definition required a DLBCL diagnosis on or prior to treatment initiation, nearly all patients had claim for cancer at baseline. ^b^The total number of patients and hospitals that are included per region on their 1L treatment in regional from Northern to Southern district of Japan.
(XLSX)

S2 Table First line treatment regimen stratified by age group. Mono: monotherapy; NOS: not otherwise specified; R: rituximab; R-CHOP: rituximab plus cyclophosphamide, doxorubicin, vincristine, and prednisone/prednisolone; R-CVP: rituximab plus cyclophosphamide, vincristine, and prednisone.
(XLSX)

S3 Table Healthcare resource utilization during the follow-up period. 1L: first-line; 2L: second-line; 3L: third-line; Dex: dexamethasone; DLBCL: diffuse large B-cell lymphoma; ER: emergency room; ICU: intensive care unit; LOS: length of stay; Mono: monotherapy; NOS: not otherwise specified; R: rituximab; R-CHOP: rituximab plus cyclophosphamide, doxorubicin, vincristine, and prednisone/prednisolone; R-CVP: rituximab plus cyclophosphamide, vincristine, and prednisone; SD: standard deviation. ^a^Immunotherapy, targeted therapy, or hormone therapy.
(XLSX)

S4 Table Direct costs in USD during the follow-up period. 1L: first-line; 2L: second-line; 3L: third-line; Dex: dexamethasone; DLBCL: diffuse large B-cell lymphoma; ER: emergency room; ICU: intensive care unit; LOS: length of stay; Mono: monotherapy; NOS: not otherwise specified; R: rituximab; R-CHOP: rituximab plus cyclophosphamide, doxorubicin, vincristine, and prednisone/prednisolone; R-CVP: rituximab plus cyclophosphamide, vincristine, and prednisone
(XLSX)
